# Comparison of Fragmentation and Dusting Modality Using Holmium YAG Laser during Ureteroscopy for the Treatment of Ureteral Stone: A Single-Center’s Experience

**DOI:** 10.3390/jcm11144155

**Published:** 2022-07-17

**Authors:** Bo-Han Chen, Tsu-Feng Lin, Chih-Chun Tsai, Marcelo Chen, Allen W. Chiu

**Affiliations:** 1 Department of Urology, MacKay Memorial Hospital, Taipei 104, Taiwan; bhchen.4392@mmh.org.tw (B.-H.C.); marcelo@mmh.org.tw (M.C.); whchiu1216@gmail.com (A.W.C.); 2 School of Medicine, MacKay Medical College, New Taipei City 252, Taiwan; 3 MacKay Junior College of Medicine, Nursing and Management, Taipei 112, Taiwan; 4 Department of Mathematics, Tamkang University, New Taipei City 251, Taiwan; 141400@mail.tku.edu.tw; 5 School of Medicine, National Yang-Ming University, Taipei 112, Taiwan

**Keywords:** dusting, energy, fragmentation, frequency, laser lithotripsy

## Abstract

Laser ureteroscopic lithotripsy (URSL) is an efficacious treatment for ureteral stones. There have been few previous studies comparing the different energy and frequency settings for URSL in a single center. We compared these two laser modalities, which were simultaneously used in our medical center for the treatment of ureteral stones. Patients who underwent fragmentation or dusting laser URSL between September 2018 and June 2020 were retrospectively reviewed. We compared patients who underwent fragmentation and dusting laser and assessed the enhancing factors for stone free rate. There were a total of 421 patients with ureteral stones who met the study criteria. There was no significant difference between the characteristics of both groups. The fragmentation group had a better stone free rate and a lower retropulsion rate compared with the dusting group. Multivariate analysis revealed that stone basket use, no upper ureteral stone or pyuria significantly improved the stone free rate. Both laser modes were effective and safe for ureteral lithotripsy although the fragmentation system showed slightly higher effectiveness and lower complication rate.

## 1. Introduction

Ureteral stones and the symptoms related to ureteral obstruction usually adversely affect the patients’ quality of life [1]. There are several therapeutic options for ureteral stones, including observation and medical expulsive therapy, shock-wave lithotripsy, ureteroscopic lithotripsy, open surgery, laparoscopic stone removal and percutaneous antegrade ureteroscopy [2,3]. The decision of which method is most suitable for each patient depends on the stone’s anatomical location and characteristics, and the safety profile of each technique [4]. Since the development of the semirigid ureteroscope with a smaller caliber, ureteroscopic lithotripsy (URSL) has become an efficacious modality for the treatment of stones in all locations in the ureter [2].

Various different energy sources can be used for lithotripsy during a ureteroscopy, including pneumatic, ultrasound, electrohydraulic and laser [3,5]. Nowadays, holmium laser lithotripsy is one of the most widely used approaches by urologists because it leads to fewer complications and has a lower incidence of upward stone migration [6,7]. 

The holmium laser breaks down the stone via the power generated from a large crystal that is coupled to fibers [8]. The power can be adjusted by the parameters of energy, frequency and pulse width [8]. The high energy, low frequency setting is defined as the fragmentation mode, while the low energy, high frequency setting is defined as the dusting mode [8,9]. The main advantage of dusting is that it produces smaller fragments and results in a shorter operation (OP) time; however, it requires a more advanced laser system which usually requires more expensive equipment [8]. Some studies thought that using the dusting laser mode could reduce the risk of ureteral injury; however, there is little evidence that one approach is better than the other [9]. 

Previous studies have compared the efficacy and safety of different energy and frequency settings, but there have been few studies comparing the use of fragmentation and dusting laser systems in a single center [10,11]. The fragmentation laser mode and the dusting laser mode were simultaneously used in our medical center, MacKay Memorial Hospital. In this study, we compared the efficacy and outcomes of these two laser modalities for the treatment of ureteral stones.

## 2. Materials and Methods

### 2.1. Study Population and Inclusion Criteria

Patients aged ≥20 years who underwent URSL for ureteral stones using a laser system in MacKay Memorial Hospital between September 2018 and June 2020, were retrospectively reviewed. This study was approved by the Institutional Review Board of Mackay Memorial Hospital as a retrospective observational cohort study. The Institutional Review Board number was 21MMHIS242e.

### 2.2. Exclusion Criteria

**Figure 1:**
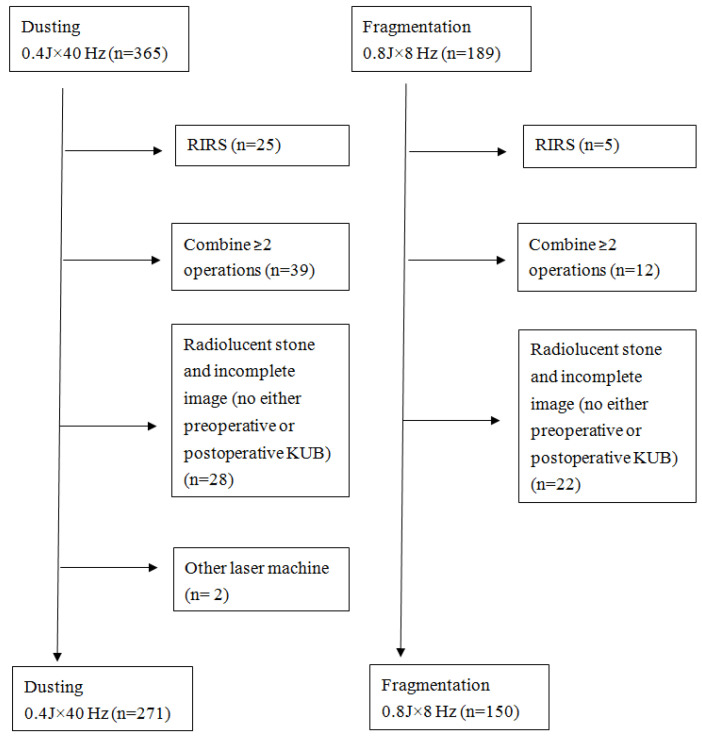
Flowchart of patient selection.

Patients with radiolucent stones, those who were lost to follow-up, and those who had incomplete image records (a lack of either preoperative images or images within 1 postoperative month) were excluded. Patients who underwent concurrent retrograde intrarenal surgery (RIRS) for concurrent renal stones or combination therapy with other treatment modalities were also excluded (Figure 1).

### 2.3. Evaluations

The patients’ age, gender, anesthesia method, admission rate, stone burden, stone number, stone laterality, stone location, ureteral condition, prevalence of pyuria, OP time, lithotripsy effectiveness, stone free rate, presence of retropulsion, secondary intervention (extracorporeal shock wave (ESWL) or URSL), and complications, including ureteral injury and postoperative ureteral stricture, were recorded. The patients’ profiles were acquired from the registration at the time they came to our hospital. The type of ureteral stent inserted was also recorded, including retrograde ureteral catheter (RUC; whistle tip ureteral catheter, Cook Medical, Bloomington, IN, USA), double J (DJ; Universa soft, Cook Medical, Bloomington, IN, USA) or stone basket (Zero Tip 2.4F, Boston Scientific, Marlborough, MA, USA).

**Figure 2:**
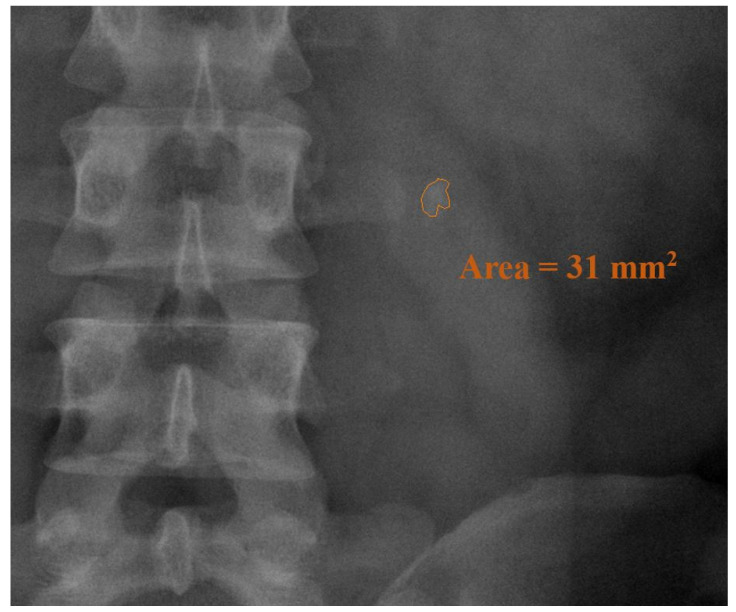
Area of the stone as calculated by the imaging system.

Depending on the patients’ condition and the attending physicians’ clinical decision, the patients were either admitted to the urology department postoperatively or they recovered from their URSL at an outpatient clinic [12]. The anesthesia method was determined by the anesthesiologist following their preoperative evaluation. Stone location and number of stones were determined from a preoperative plain radiography of the kidney, ureter, and bladder (KUB). Stone burden was defined as the area of the stone calculated by our imaging system after the stone was delineated (Figure 2). Lithotripsy effectiveness was defined as the stone burden divided by the OP time (mm^2^/min). Pyuria was defined as >10 white blood cells in the preoperative urine analysis under a high power field. The stone’s retropulsion, ureter condition, and postoperative injury were documented according to the surgeons’ intraoperative findings. Follow-up plain radiography KUB or computerized tomography (CT) were performed within 1 month of the URSL. The definition of stone free is not standardized and differs greatly in previous literature [9]. In the current study, stone-free was defined as no residual stones > 3 mm in the ureter of the affected side within 1 postoperative month [13]. We searched the medical records for any complications and secondary interventions, including a repeat URSL, ESWL or RIRS for stones that remained in the ureter or were pushed back to the renal calyx during the initial operation. 

### 2.4. Procedures

A semirigid 6/7.5-F ureteroscope (Wolf, Knittlingen, Germany) and a Full HD 3CCD camera head system (CH-S190-XZ-E, Olympus, Tokyo, Japan) were used for all procedures. All URSL operations were performed by the same experienced urology team for both modes. The initial settings for the fragmentation laser system (Auriga XL 50W, Boston Scientific, Marlborough, MA, USA) with a 365 μm fiber were: energy 0.8 Joules and frequency 8 Hertz. The initial settings for the dusting laser system (VersaPulse 60W, Lumenis, Yokneam, Israel) with a 365 μm fiber were: energy 0.4 Joules and frequency 40 Hertz. The patient was placed in a reverse Trendelenburg position and the intraluminal water pressure was decreased by lowering the water bottle. After the lithotripsy was finished, the larger stone fragments were removed using a retrieval basket and the smaller ones were left for spontaneous passage. At the end of the procedure, a ureteral stent was considered, depending on the fragmentation status of the residual stones, the ureteral condition, bleeding, and the index of suspicion for postoperative ureteral stricture. Perioperative intravenous broad spectrum antibiotics were given to all patients and oral antibiotics were given to outpatients postoperatively. Postoperative follow-up was arranged for each patient in the same urology team.

### 2.5. Statistical Analysis

A *t*-test, Chi-squared test and Fisher’s exact test were used for statistical analysis, and the statistical significance was set at *p* < 0.05. SPSS for Windows (version 22.0; IBM SPSS Statistics, IBM Corporation, Chicago, IL, USA) was used to perform the logistic regression analysis of risk factors for stone free rate. Propensity score matching (PSM) analysis was used to adjust for the differences between the two groups, thus helping to make the results as accurate as possible. Matching was performed using the “matchit” package in R (Vienna, Austria, version 3.0.2) for basket usage, type of anesthesia, admission, gender, laterality, stone number, stone location, ureteral condition and pyuria. A total of 150 patients each were selected after matching. The *t*-test, Chi-squared test and Fisher’s exact test were used to explore the differences between the fragmentation and dusting groups. All tests were two-sided, and *p* values < 0.05 were considered to be statistically significant.

## 3. Results

A total of 421 patients were analyzed, including 271 who received dusting laser URSL and 150 who received fragmentation laser URSL. The mean age was 54.1 ± 12.63 years in the dusting group and 53.95 ± 13.57 years in the fragmentation group. The mean stone burden was 56.8 ± 60.71 mm^2^ in the dusting group and 48.9 ± 59.23 mm^2^ in the fragmentation group. There were no significant differences in gender, age, admission rate, anesthesia methods, stone laterality, stone location and ureteral condition between the two groups. However, there were significantly more patients in the dusting group that had multiple ureteral stones (*p* = 0.023) and pyuria (*p* < 0.001) compared with the fragmentation group (Table 1).

After 1:1 PSM analysis, there are 150 patients in each group. There was no significant difference between the characteristics of dusting and fragmentation groups. The mean age was 53.23 ± 12.68 years and the stone burden was 51.33 ± 58.69 mm^2^ in the dusting group (Table 2). 

The treatment results revealed that the dusting group had a significantly higher retropulsion rate (*p* = 0.022) compared with the fragmentation group. The dusting group had a slightly lower stone free rate (*p* = 0.159) compared with the fragmentation group. However, although the dusting group had higher retropulsion rate and lower stone free rate, the secondary intervention rate in dusting group was just lower (*p* = 0.115) than the fragmentation group. There were no statistically significant differences in the OP time, effectiveness, insertion rate for both types of ureteral stents, stone basket usage rate, ureteral injury rate and complication rate between the two groups (all *p* > 0.05) (Table 3). 

Complications included three cases of bleeding or oozing during the OP, one case of postoperative prostatitis, one case of leg dropping from the lithotomy position and four cases of postoperative ureteral strictures. The three patients with intraoperative bleeding or oozing had a prolonged OP time for a hemostasis procedure. The patient who suffered from prostatitis was diagnosed by CT image, admitted again 5 days after the URSL and received intravenous antibiotics for 10 days. The patient with one leg dropping from the lithotomy position received fluoroscopic imaging and there was no fracture or dislocation. Among the four patients with postoperative ureteral stricture, one of them had laser ureterotomy, and three of them did not need further treatment. 

Univariate analysis showed that patients who were stone free were slightly more likely to have received the fragmentation laser system (95% confidence interval [CI]: 0.85–2.61, odds ratio [OR] = 1.49, *p* = 0.16), and had significantly higher stone basket usage rate (95% CI: 2.56–8.4, OR = 4.63, *p* < 0.001), fewer upper ureteral stones (95% CI: 1.52–84.39, OR = 11.34, *p* = 0.018) and less pyuria (95% CI: 1.58–5.25, OR = 2.87, *p* = 0.001) compared with patients who were not stone free (Table 4). Further, the significant items in univariate logistic analysis were selected to do multivariate analysis. Multivariate logistic analysis revealed that patients who were stone free had a significantly higher stone basket usage rate (95% CI: 2.14–7.24, OR = 3.93, *p* < 0.001), fewer upper ureteral stones (95% CI: 1.06–61.67, OR = 8.1, *p* = 0.044) or less pyuria (95% CI: 1.18–4.25, OR = 2.25, *p* = 0.013) compared with those patients who were not stone free (Table 4).

## 4. Discussion

Due to advances in technology, there are now a wide variety of power generators that can be used for ureteroscopic lithotripsy. Chen et al. reported that laser lithotripsy is superior to pneumatic lithotripsy for the management of ureteral stones in terms of the stone free rate and the secondary intervention rate for stones of all sizes, at their institute [13]. Yin et al. also found that laser lithotripsy had advantages over pneumatic lithotripsy, with a high stone-free rate and a low migration rate in their meta-analysis [6]. If properly used, the laser generator provides a highly effective tool for disintegrating stones in the urinary tract with minimal additional safety concerns [14]. Furthermore, there have been large improvements in the laser technique. In our study, we found that the laser mode had no significantly related to stone free rate. Therefore, we believe that both of these techniques are effective and safe for ureteral lithotripsy. However, because some of these technologies are relatively novel, more comprehensive studies may be needed to confirm the efficacy and safety of these innovations.

Among the different types of laser sources, holmium: yttrium-aluminum-garnet (Ho:YAG) is the most popular source for URSL because of its ability to fragment stones of any composition and its excellent safety profile [15]. The laser power of Ho:YAG can be highly absorbed in water and has a low penetration depth; therefore, Ho:YAG induces little injury to the surrounding tissue [8]. The laser is generated and releases its energy from the tip of a fiber. The stones are then disintegrated into fragments or dust [8]. The pulse energy settings for the holmium laser usually range from 0.2 to 2.0 Joules and the frequency setting usually ranges from 4 to 80 Hertz depending on the manufacturer and the model of laser console used [9]. Different settings may cause diverse effects on the stones. The higher the pulse energy used, the more stone retropulsion occurs, whereas changes in frequency had a minimal effect on retropulsion when the energy and fiber diameter were kept constant [16]. In our study, the dusting group had a significantly higher stone retropulsion rate compared with the fragmentation group. However, the high push back rate in the dusting group did not cause a significantly lower stone free rate or higher secondary intervention rate. We thought this maybe implied that the retro-pulsed stones were insignificant, did not need secondary intervention, and did not affect the stone free rate much. Furthermore, in the logistic regression analysis, the laser mode was also not significantly related to the stone free rate (*p* = 0.16). We hypothesized that the high retropulsion rate could be due to the diversity of the patients’ characteristics, especially pyuria and spinal anesthesia, which could hinder the URSL procedure and its outcomes. Senocak et al. found that patients with fever and pyuria had a significantly higher risk of postoperative infectious complications [17]. Surgeons usually try to use the least OP time to treat patients with pyuria to avoid postoperative sepsis. Rushed operations can cause a high push back rate although the surgeons were all experienced. Kizilay et al. also found that the rate of push-back to the collecting system was higher in the spinal anesthesia group compared to the general anesthesia group from their multi-center study group [18]. They thought that the relaxed ureter would be a factor that makes the stone more mobile and facilitates the push-back of the stone. In our data, there were few patients with pyuria and spinal anesthesia in the dusting group.

Although URSL has become a common surgery, its associated risk of urinary tract infection is about 25% in patients without prophylactic antibiotics [19]. To prevent patients from postoperative urosepsis, surgeons need to accomplish the URSL as quickly as possible, particularly for patients with pyuria [20]. If the patient is simultaneously suffering from a severe infection, the surgeon should break through the obstructive ureteral stone rapidly and place a ureteral stent for emergent drainage of the renal pelvis, instead of crushing the stone into smaller pieces. Under these circumstances, the stone free rate can be decreased, and in the current study, patients with pyuria had a significantly lower stone free rate. On the other hand, compared with solitary stones, multiple stones can also decrease the stone free rate in lithotripsy surgery [21]. In our study, single stones were slightly prone to have a higher stone free rate. Stone location also has a significant effect on stone clearance. Degirmenci et al. stated that the stone clearance rate was higher for lower ureteral stones compared with upper ureteral stones [22]. In our study, patients without upper ureteral stones showed a significantly higher stone free rate compared with stones in other locations. Furthermore, a review by Brain et al. found that using a stone basket was associated with a more complete initial stone clearance [9]. We also found similar results, as URSL with basket use had a higher stone free rate compared with URSL without basket use.

The current study had several limitations. First, this was a retrospective study, which means there was a patient selection bias, including some significant differences in the patient characteristics. Second, we did not set a standard follow-up duration for all of the patients. Our postoperative follow-up time depended on the patient’s symptoms and their willingness to engage with clinicians. Asymptomatic patients tended to have a longer follow-up time compared with patients who suffered from immediate symptoms. Third, we did not do a complete analysis of the stone composition, which could have influenced the effectiveness of lithotripsy. However, in Asia, calcium oxalate is the most frequent component of stones and it accounts for about 90% of all calculi in the urinary tract [23]. Therefore, the bias caused by stone composition may not have a large effect. Fourth, we used two different machines for the two different laser settings. There may be more bias between the two machines than if the two different settings were used on the same machine. Finally, not all of the URSL in the present study were performed by the same urologist. However, all the urologists involved in this study had achieved the same qualification. In other words, there was only a small operating difference between each surgeon in the urologist team.

## 5. Conclusions

In conclusion, both laser modes were effective and safe for ureteral lithotripsy. We suggested the fragmentation system for ureteral stones since it showed slightly higher effectiveness and a lower complication rate. The energy and frequency settings did not have a significant effect on the stone free rate in these two modalities. Enhancing factors for a higher stone free rate included operation with the use of a stone basket (OR = 3.932), no upper ureteral stone location (OR = 8.095) or pyuria (OR = 2.245). To achieve higher stone free rate, using a stone basket is recommended.

## Figures and Tables

**Table 1:** Patient characteristics compared between the dusting and fragmentation groups.

Variables		Dusting Group (*n* = 271)	Fragmentation Group (*n* = 150)	*p*-Value
Female/Male (Female %)		114/157 (42.1)	50/100 (33.3)	0.078
Left/Right (Left %)		151/120 (55.7)	84/66 (56)	0.956
Admission rate, *n* (%)		206 (76)	125 (83)	0.079
Mean age, year; mean ± SD *		54.1 ± 12.63	53.9 ± 13.57	0.89
Mean stone burden, mm^2^; mean ± SD *		56.8 ± 60.71	48.9 ± 59.23	0.199
Anesthesia, *n* (%)				0.312
	Spinal	102 (38)	64 (43)	
	General	169 (62)	86 (57)	
Number of stones, *n* (%)				0.023
	Single	219 (81)	134 (89)	
	Multiple	52 (19)	16 (11)	
Stone location, *n* (%)				
	Upper	234 (86)	135 (90)	0.275
	Middle	25 (9)	12 (8)	0.671
	Lower	20 (7)	10 (7)	0.785
Ureter condition, *n* (%)				
	Polyposis	55 (20)	22 (15)	0.153
	Angulation	40 (15)	26 (17)	0.487
	Stricture	32 (12)	23 (15)	0.304
	Edema	22 (8)	5 (3)	0.055
Pyuria, *n* (%)		110 (41)	33 (22)	<0.001

* Abbreviations: SD, standard deviation.

**Table 2:** Patient characteristics compared between the dusting and fragmentation groups after matching.

Variables ^1^		Dusting Group (*n* = 150)	Fragmentation Group (*n* = 150)	*p*-Value
Female/Male (Female %)		101/49 (67)	100/50 (67)	0.902
Left/Right (Left %)		81/69 (54)	84/66 (56)	0.728
Admission, *n* (%)		125 (83)	125 (83)	1
Mean age, year; mean ± SD ^2^		53.23 ± 12.68	53.95 ± 13.57	0.638
Mean stone burden, mm^2^; mean ± SD ^2^		51.33 ± 58.69	48.9 ± 59.24	0.722
Anesthesia, *n* (%)				0.816
	Spinal	66 (91)	64 (89)	
	General	84 (9)	86 (11)	
Number of stones, *n* (%)				0.558
	Single	137 (91)	134 (89)	
	Multiple	13 (9)	16 (11)	
Stone location, *n* (%)				
	Upper	128 (85)	135 (90)	0.219
	Middle	12 (8)	12 (8)	1
	Lower	9 (6)	10 (7)	0.813
Ureter condition, *n* (%)				
	Polyposis	24 (16)	22 (15)	0.749
	Angulation	25 (17)	26 (17)	0.878
	Stricture	26 (17)	23 (15)	0.639
	Edema	1 (1)	5 (3)	0.214
Pyuria, *n* (%)		35 (23)	33 (22)	0.783

^1^ Propensity score matching was carried out by basket usage, type of anesthesia, admission, gender, laterality, stone number, stone location, ureteral condition and pyuria. ^2^ Abbreviation: SD, standard deviation.

**Table 3:** Comparison of outcomes for lithotripsy between the dusting and fragmentation groups after matching.

Variables		Dusting Group (*n* = 150)	Fragmentation Group (*n* = 150)	*p*-Value
OP ^1^ time, min; mean ± SD ^2^		37.73 ± 17.92	37.6 ± 19.14	0.95
Effectiveness, mm^2^/min; mean ± SD ^2^		1.37 ± 1.16	1. 45 ± 1.56	0.613
Ureteral stent insertion, *n* (%)		128 (85)	127 (85)	0.872
	RUC ^3^	7 (5)	9 (6)	0.607
	DJ ^4^	121 (81)	118 (79)	0.667
Basket use, *n* (%)		87 (58)	93 (62)	0.48
Stone free, *n* (%)		113 (75)	123 (82)	0.159
Retropulsion, *n* (%)		29 (20)	15 (10)	0.022
Secondary intervention, *n* (%)		19 (18)	29 (19)	0.115
Ureter injury, *n* (%)		0 (0)	1 (1)	0.5
Complication, *n* (%)		6 (4)	3 (2)	0.501

^1^ Abbreviation: OP, operation; ^2^ Abbreviation: SD, standard deviation; ^3^ Abbreviation: RUC, retrograde ureteral catheter; ^4^ Abbreviation: DJ, double J.

**Table 4:** Univariate and multivariate logistic regression analysis of the stone free rate after matching.

Variables	Univariate				Multivariate			
	*p*-Value	OR^1^	95% CI ^2^ of OR ^1^		*p*-Value	OR ^1^	95% CI ^2^ of OR ^1^	
Fragmenation system	0.16	1.492	0.854	2.606				
Basket use	<0.001	4.632	2.555	8.397	<0.001	3.932	2.136	7.238
General anesthesia	0.136	1.525	0.876	2.656				
Female gender	0.737	1.107	0.611	2.004				
Right ureter stone	0.428	1.254	0.716	2.197				
Single stone	0.074	2.115	0.93	4.81				
No upper ureteral stone	0.018	11.34	1.524	84.387	0.044	8.095	1.063	61.667
No pyuria	0.001	2.877	1.577	5.249	0.013	2.245	1.184	4.254

^1^ Abbreviation: OR, odds ratio; ^2^ Abbreviation: CI, confidence interval.

## Data Availability

Records and data pertaining to this study are in the patient’s secure medical records in Mackay Memorial Hospital and are available from the corresponding author on reasonable request.
